# Correction to “Overexpression of ACE2 ameliorates Aβ‐induced blood–brain barrier damage and angiogenesis by inhibiting NF‐κB/VEGF/VEGFR2 pathway”

**DOI:** 10.1002/ame2.12489

**Published:** 2024-08-23

Xueling Zhang, Yu Zhang, Ling Zhang*, Chuan Qin*, *Anim Models Exp Med*. 2023;6:237–244.

In Figure 2A, the picture of immune blot for GAPDH was misplaced. The correct picture has been added to the updated Figure 2A.

**FIGURE 2:**
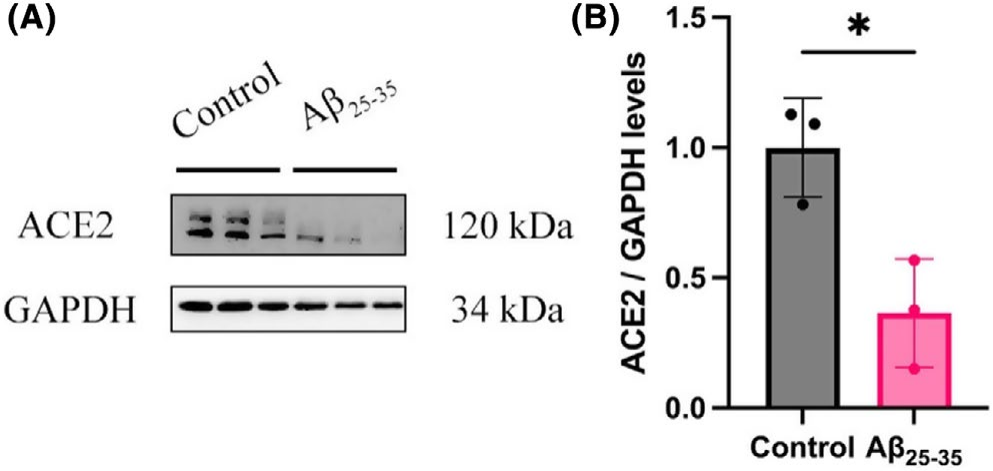
Aβ_25‐35_ treatment downregulates ACE2 expression in bEnd.3 cell. (A) Western blot analysis showing the expression levels of ACE2. GAPDH levels were measured to confirm that equal amounts of protein were loaded. (B) Histogram quantifying the results shown in (A). The data are represented as means ± SD for three replicates. *p < 0.05.

In figure 6A, the picture of immune blot for GAPDH was misplaced and the molecular weight of VEGFa was mistakenly marked. The correct picture has been added to the updated Figure 6A.

**FIGURE 6:**
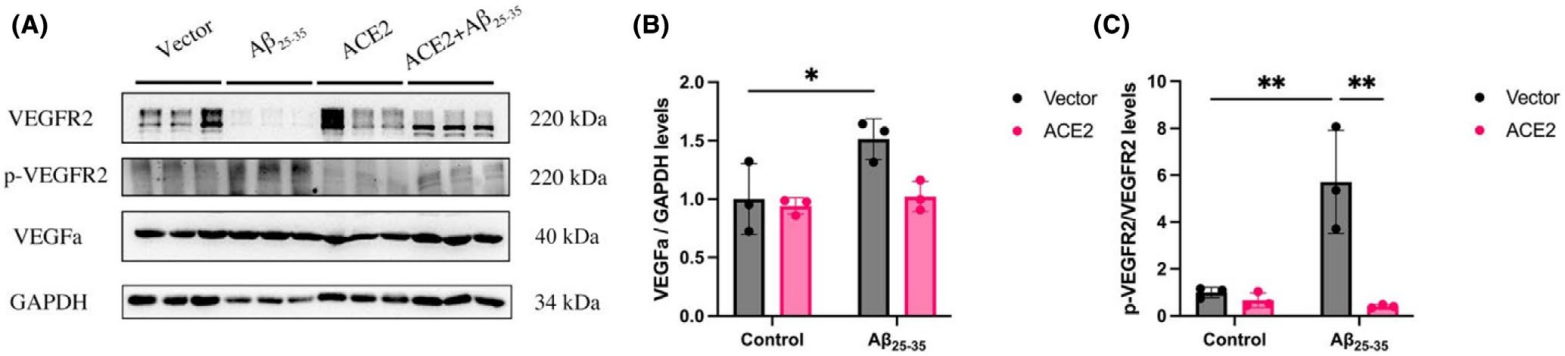
ACE2 inhibits the activation of the VEGF pathway induced by Aβ_25‐35_. (A) Western blot analysis showing the expression levels of VEGFR2, p‐VEGFR2, and VEGFa. GAPDH levels were measured to confirm that equal amounts of protein were loaded. (B) Histogram quantifying the results for VEGFa shown in (A). (C) Histogram quantifying the results for the ratio of phosphorylated VEGFR2 shown in (A).

In Figure 8A, the picture of immune blot for GAPDH was misplaced. The correct picture has been added to the updated Figure 8A.

**FIGURE 8:**
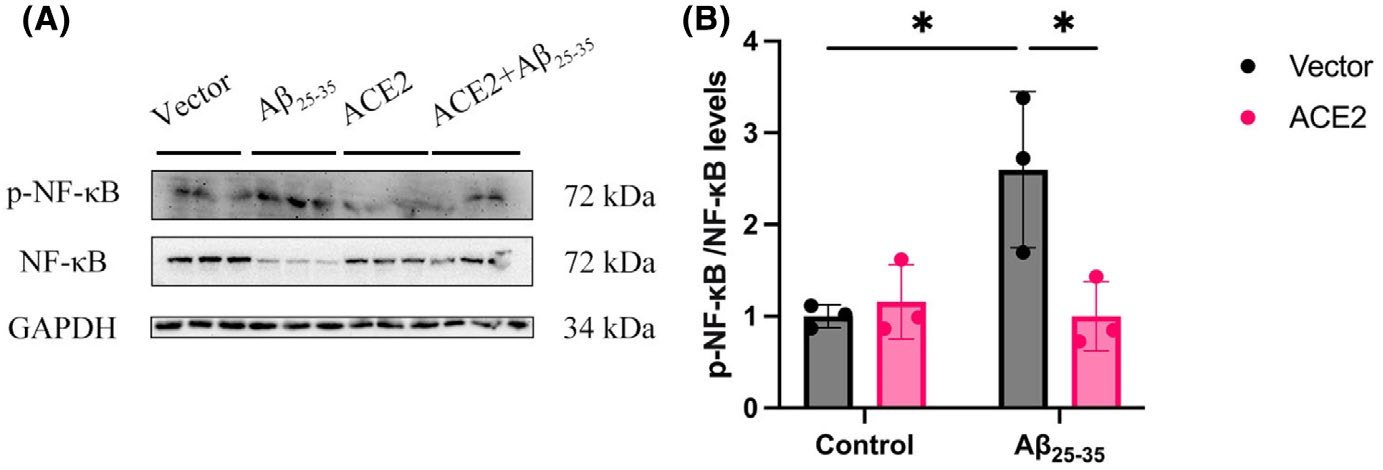
ACE2 inhibits the activation of the NF‐κB pathway induced by Aβ_25‐35_. (A) Western blot analysis showing the expression levels of pNF‐κB and NF‐κB. GAPDH levels were measured to confirm that equal amounts of protein were loaded. (B) Histogram quantifying the results for the ratio of phosphorylated NF‐κB shown in (A). *p < 0.05.

We apologize for these errors.

